# Consensus-Derived Quality Performance Indicators for Neuroendocrine Tumour Care

**DOI:** 10.3390/jcm8091455

**Published:** 2019-09-12

**Authors:** Braden Woodhouse, Sharon Pattison, Eva Segelov, Simron Singh, Kate Parker, Grace Kong, William Macdonald, David Wyld, Goswin Meyer-Rochow, Nick Pavlakis, Siobhan Conroy, Vallerie Gordon, Jonathan Koea, Nicole Kramer, Michael Michael, Kate Wakelin, Tehmina Asif, Dorothy Lo, Timothy Price, Ben Lawrence, on behalf of the Commonwealth Neuroendocrine Tumour Collaboration (CommNETs)

**Affiliations:** 1 Discipline of Oncology, Faculty of Medicine and Health Sciences, The University of Auckland, Auckland 1023, New Zealand; b.woodhouse@auckland.ac.nz (B.W.); kate.parker@auckland.ac.nz (K.P.); 2 Department of Medicine, University of Otago, Dunedin 9016, New Zealand; sharon.pattison@otago.ac.nz; 3 Department of Medical Oncology, Monash University and Monash Health, Melbourne 3800, Australia; eva.segelov@monash.edu; 4 Department of Medical Oncology, Sunnybrook Odette Cancer Center, University of Toronto, Toronto, ON M4N 3M5, Canada; simron.singh@sunnybrook.ca; 5 Department of Nuclear Medicine, Peter MacCallum Cancer Centre, Melbourne 3000, Australia; grace.kong@petermac.org; 6 Department of Nuclear Medicine, Fiona Stanley Hospital, Perth 6150, Australia; william.macdonald@health.wa.gov.au; 7 Department of Medical Oncology, Royal Brisbane and Women’s Hospital, Brisbane 4029, Australia; david.wyld@health.qld.gov.au; 8 School of Medicine, University of Queensland, Brisbane 4072, Australia; 9 Department of General Surgery, Waikato Hospital, Hamilton 3204, New Zealand; win.meyer-rochow@waikatodhb.health.nz; 10 Department of Medical Oncology, Royal North Shore Hospital, Sydney 2065, Australia; nick.pavlakis@sydney.edu.au; 11 Unicorn Foundation New Zealand, Auckland, New Zealand; siobhan@unicornfoundation.org.nz; 12 Department of Medical Oncology, Cancer Care Manitoba, Winnipeg, MB R3E 0V9, Canada; vgordon1@cancercare.mb.ca; 13 Department of General Surgery, North Shore Hospital, Auckland 0620, New Zealand; jonathan.koea@waitematadhb.govt.nz; 14 Department of Pathology, North Shore Hospital, Auckland 0620, New Zealand; nicole.kramer@waitematadhb.govt.nz; 15 Department of Medical Oncology, Peter MacCallum Cancer Centre, Melbourne 3000, Australia; michael.michael@petermac.org; 16 Unicorn Foundation, Melbourne, Australia; kate.wakelin@unicornfoundation.org.au; 17 Department of Medical Oncology, Saskatchewan Cancer Agency, Saskatoon, SK S4W 0G3, Canada; tehmina.asif@saskcancer.ca; 18 Department of Medical Oncology, St Joseph’s Health Care, Toronto, ON M6R 1B5, Canada; dlo@stjoestoronto.ca; 19 Department of Medical Oncology, The Queen Elizabeth Hospital, Adelaide 5011, Australia; timothy.price@health.sa.gov.au; 20 Department of Medical Oncology, Auckland City Hospital, Auckland 1023, New Zealand

**Keywords:** quality performance indicators, QPIs, cancer care, neuroendocrine tumour, NETs, modified Delphi, CommNETs

## Abstract

Quality performance indicators (QPIs) are used to monitor the delivery of cancer care. Neuroendocrine tumours (NETs) are a family of individually uncommon cancers that derive from neuroendocrine cells or their precursors, and can occur in most organs. There are currently no QPIs available for NETs and their heterogeneity makes QPI development difficult. CommNETs is a collaboration between NET clinicians, researchers and advocates in Canada, Australia and New Zealand. We created QPIs for NETs using a three-step consensus process. First, a multidisciplinary team used the nominal group technique to create candidates (*n* = 133) which were then curated into appropriateness statements (62 statements, 44 sub-statements). A two-stage modified RAND/UCLA Delphi consensus process was conducted: an online survey rated the statement appropriateness then the top-ranked statements (*n* = 20) were assessed in a face-to-face meeting. Finally, 10 QPIs met consensus criteria; documentation of primary site, proliferative index, differentiation, tumour board review, use of a structured pathology report, presence of distant metastasis, 5- and 10-year disease-free and overall survival. These NET QPIs will be trialed as a method to monitor and improve care for people with NETs and to facilitate international comparison.

## 1. Introduction

Evidence-based quality performance indicators (QPIs) are used to improve quality of cancer care by recording and publishing key aspects of each individual patient’s cancer journey that contribute to outcome. For example, colorectal cancer QPIs include stoma-free survival, tumour board review, and the use of adjuvant chemotherapy [1]. QPI measurement can identify under-performing centres, and also indirectly provide standards that a service can aspire to [2,3,4,5,6,7,8]. QPIs have been developed in multiple countries for common malignancies such as breast or bowel cancer [1,9,10,11,12], and are usually selected from an evidence base of factors associated with outcome. This type of data are more often available in cancers that are common, have a single organ of origin (e.g., breast), and a predominant histology (e.g., adenocarcinoma). For example, separate sets of QPIs have been developed and implemented in Canada, Australia and New Zealand for colorectal cancer [1,13,14,15,16,17].

Neuroendocrine tumours (NETs) are a family of malignancies that derive from neuroendocrine cells or their precursors. NETs most commonly arise in the gastrointestinal tract [18], the lung and also occur in endocrine organs, thymus, skin, and all organs of the genito-urinary and gynaecological systems. Some NETs release hormones which in excess lead to specific symptoms, such as flushing and diarrhoea caused by excess serotonin secretion, or hypoglycemia caused by excess insulin, for example. Although NETs are uncommon (incidence 6.98 per 100,000), their incidence is rising [19,20,21,22], and because some NETs are very slow growing the prevalence is higher than other cancers of the same location; for example gastrointestinal NETs have a higher prevalence than pancreatic and gastric carcinomas [23].

NET outcome is highly variable by grade, usually described by proliferative index (Ki-67 and/or mitotic count), and the pace of progression varies from extremely rapid to very slow, with survival in the metastatic setting ranging from weeks to decades. Presentation will also vary due to functional status of the tumour and secreted hormone(s). This variability matters in the clinic; for example, the 5-year overall survival of rectal NETs is over 85%, whereas pancreatic NETs is less than 40% [21], and the treatments required for NETs from each site is mostly distinct. The biological heterogeneity, socioeconomic factors and regional variations of clinical care also present challenges to the treating clinical team, and present difficulties for appraising the quality of care of people with NETs within, and across health care systems [24]. QPIs that measure fundamental aspects of NET diagnosis and treatment outcome could be used to monitor the quality of NET care.

NETs present a challenge for QPI development. A QPI strategy for NETs must balance a tension between measuring fundamentals that underlie the care of all types of NETs, yet still enable detection of the variability inherent in different NET subtypes. Some pathologies that must be detected in people with NETs are very rare, thus questioning their value as a general indicator of quality care. An example is detection of carcinoid heart disease, where only a small fraction of people with NETs are affected. Types of indicators are also influenced by different healthcare systems, because data collection, regulatory processes and treatment options vary between jurisdictions. Countries like New Zealand (NZ), Canada and Australia have predominantly publically funded health care systems with some degree of centralised health data collection. For example, the NZ government collects data within a national cancer registry which includes a minimum dataset describing each cancer, alongside mortality, hospital billing information, and data on prescription of pharmaceuticals; similar data exist in some provinces in Canada, and in some states in Australia.

CommNETs is an international collaboration of NET clinicians, researchers and advocates from Australia, NZ and Canada with a mandate to accelerate research in NETs and improve NET care. The need for NET QPIs was identified as a means to monitor and standardise comparisons in order to improve outcomes.

## 2. Methods

**Figure 1:**
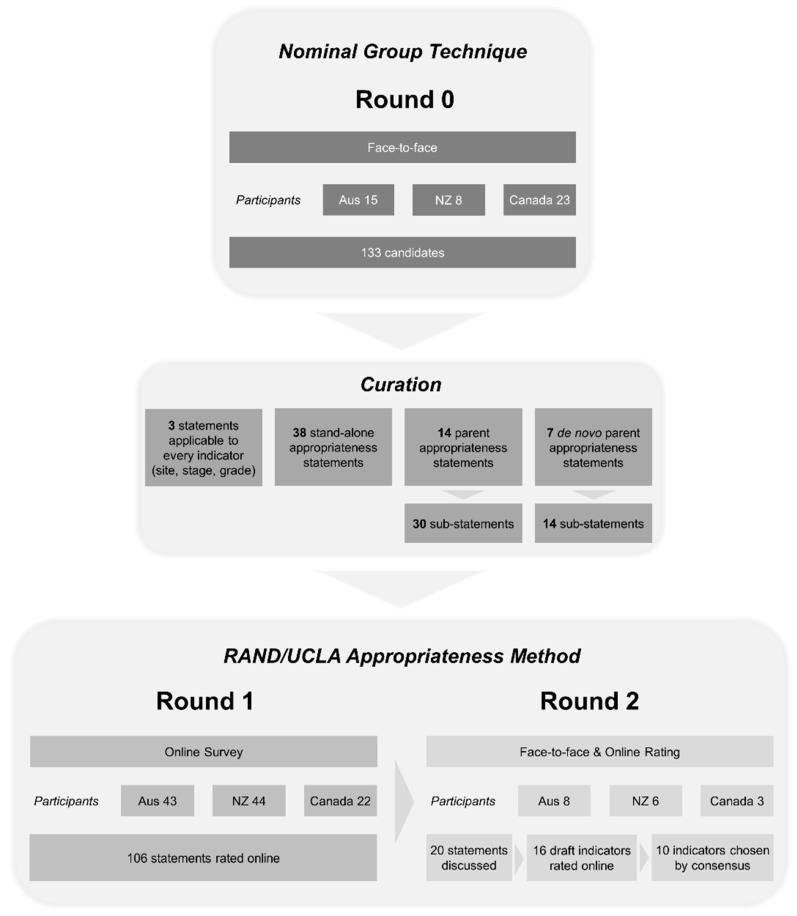
Summary of method. Nominal group technique (NGT) was used to generate 133 “candidates” for neuroendocrine tumour (NET) quality performance indicators (QPIs) (Round 0). These candidates were converted into 106 “appropriateness statements.” In Round 1 these statements were evaluated using the RAND/UCLA Appropriateness Method. Participants rated the importance and measurability of each statement as indicators of care quality in an online survey, which led to 20 statements being selected for further discussion. In Round 2, a small group of experts discussed these 20 statements, rejected some, and rated the remainder online, leading to a final list of 10 QPIs.

The original process plan used a two-round modified Delphi consensus (RAND/UCLA Appropriateness Method) to select NET-specific QPIs from the QPI literature [25]. However, a literature search returned no relevant results (search strategy Supplementary Material S1). The method was adjusted to include an initial step for generation of candidates for NET QPIs, and is summarised in Figure 1. The first phase (Round 0) aimed to formulate as many measures of NET patient care quality as seen to be relevant. Next, a small group of experts curated the items and made them unambiguous and appropriate for evaluation. Then, Round 1 included a large number of participants from varying disciplines who rated the statements’ importance and measurability, in order to identify the top statements. Finally, Round 2 consisted of an expert panel (primarily NET clinicians), who met in person to evaluate the top-ranked statements and provide a final rating.

### 2.1. Participants

Participants were drawn from the three CommNETs countries (NZ, Canada, Australia), and were multidisciplinary in background, including patients and their advocates (see Figure 1; see Supplementary Material S2).

### 2.2. Round 0—The Generation of Candidate Statements

Nominal group technique (NGT) is a structured method for idea generation that encourages balanced individual participation; chosen to ensure that the voice of patient advocates and non-clinical disciplines would be heard. NGT was used as previously described [26] and is further summarised in Supplementary Material S3. Participants were allocated into six groups pre-selected to include a range of nationality and multidisciplinary expertise. They received an education session including a background to the project, definitions of QPIs, and the NGT method. Groups generated ideas for NET-specific QPIs for four phases of the NET patient journey. The top five ideas from each phase were taken forward as ‘candidates’ for assessment in the consensus process. Group membership was changed regularly to encourage new interaction.

### 2.3. The Conversion of Candidate Statements into Appropriateness Statements

According to the methodology of the modified RAND/UCLA process, “candidates” were converted into statements so that their appropriateness as NET QPIs could be rated. Each appropriateness statement began with the candidate (e.g., *Survival after diagnosis*…) followed by the phrase “…*is an important and measurable indicator of NET care quality*”. For example, the candidate ‘patient reported quality of life’ becomes the statement ‘patient reported quality of life is an important and measurable indicator of NET care quality.’ Some candidates required modification to become ‘appropriateness statements’. This curation step (conducted by BL, BW and SP) used the following criteria: candidates with more than one variable or time point were separated into multiple single appropriateness statements; duplicate candidates were discarded; candidates that included multiple concepts were excluded, and candidates with ambiguous statements had additional words added for clarity, with care taken to enhance the intended meaning.

Three factors were repeatedly included in multiple Round 0 candidates; namely site, stage and grade. For example, the candidate “5-year survival by *site*, *stage and grade*” contains multiple components for ranking, and acknowledges that QPIs vary by site, stage and grade. These three factors were separated and are hereon in referred to as “core indicators.” These were presented individually and participants asked whether each was “…*required to robustly interpret each indicator of NET care quality*”. Respondents were, therefore, asked to evaluate the necessity of these core indicators to other indicators, and not their individual importance and measurability.

### 2.4. Round 1—Online Survey

Survey Monkey^®^ was used to present appropriateness statements and record ratings and feedback. Participants separately rated the *importance* of each statement, and the *measurability* of each statement (see Supplementary Material S4). Participants used a Likert scale to rate each statement from highly inappropriate “1”, to uncertain “5”, to highly appropriate “9”. A weighted average was calculated for positive responses (6–9) using the number of participants and the rating allocated (See Supplementary Material S5). Only responses from participants who completed all fields were included. We arbitrarily determined that statements would be considered important, and measurable, if the positive weighted average was greater than three for both scores.

### 2.5. Round 2—Modified RAND/UCLA Delphi Consensus Expert Group Ranking

As required by the modified Delphi method, a small expert panel (see Supplementary Material S2, Tables S2 and S3) met to discuss appropriateness statements that had been top ranked in the Round 1 survey, and select a subset of final indicators by consensus. Following the meeting, a rating form was circulated online for rating the draft indicators by the expert group as appropriate, uncertain, or inappropriate. Final NET QPIs were chosen using a consensus threshold of 80%, as utilised in the previous CommNETs Delphi process [27] (see Supplementary Material S6).

## 3. Results

The number of participants, candidates and appropriateness statements are summarised in Figure 1. Round 0 included 46 multidisciplinary participants (Medical Oncology, Surgery, Endocrinology, Radiation Oncology, Nuclear Medicine, Pathology, Radiology, Research, Pharmacy, Nursing, Patients and their advocates) who produced 133 candidates. Conversion into appropriateness statements required separation of candidates with more than one time point into multiple single statements; duplicates discarded; indicators with multiple concepts excluded, and ambiguity clarified. Statements were organised using a hierarchical structure using appropriateness statements and sub-statements (see Figure 1 and Supplementary Material S3).

**Figure 2:**
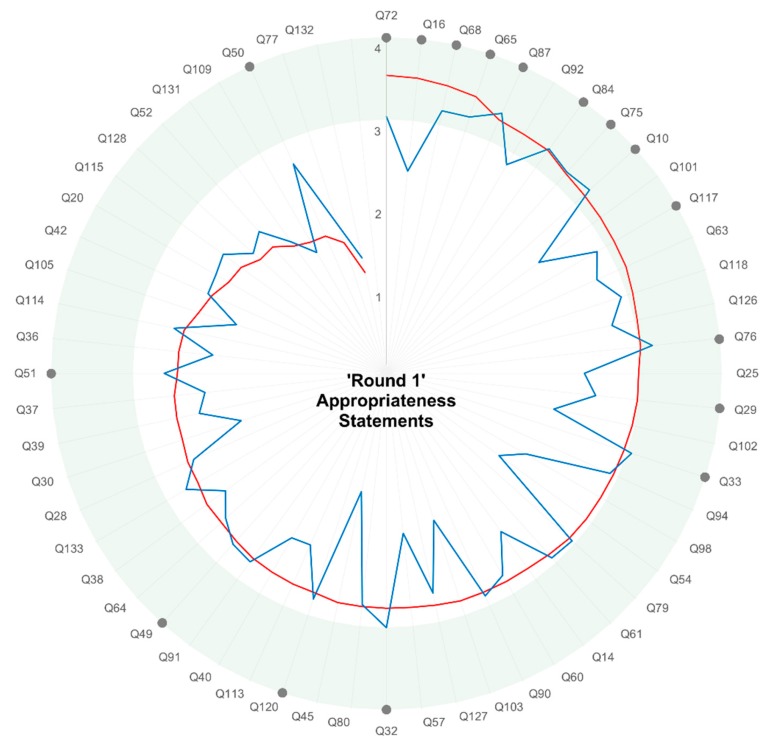
Round 1 ranking of appropriateness statements, ordered by importance. Each radial spoke represents a Round 1 appropriateness statement. Weighted averages for ratings of “Importance” (red line) and “Measurability” (blue line) are presented. Many statements rated as important were not measurable, and a few measurable indicators were not rated as important. Statements taken forward to Round 2 are shown by grey dots, and tended to be both important and measurable (>3 shaded in green). The wording of each statement (e.g., Q72) is shown in Supplementary Material S7.

The Round 1 survey was sent to 237 people. There were 109 responders, and 71 participants completed all fields in the survey. As some participants sent on the questionnaire to others in their own NET clinical communities, we are unable to calculate an overall response rate. The rating of appropriateness statements in Round 1 showed variable importance (min 1.2, max 3.5) and measurability (min 1.4, max 3.4). Eight statements that were rated as important were not considered measurable. The ratings of the 59 appropriateness statements from Round 1 (after removal of the three core statements, whose importance and measurability were not directly rated) are shown in Figure 2 in order of the weighted average of ‘importance’ (see Supplementary Material S7 for the corresponding statements). Eight statements (and 13 sub-statements) were rated as both important and measurable (Figure 2). Nine statements (and four sub-statements) were important but not measurable. Forty-two statements (and 17 sub-statements) rated neither important nor measurable.

The small expert group (*n* = 17) met in Round 2 to discuss appropriateness statements indicated by grey dots in Figure 2. This included statements rated both important and measurable in Round 1. The Round 1 ranking methodology could exclude statements because of their wording rather than the value of the concept they described, so several lower-ranked statements were brought forward for ‘last chance’ discussion by Round 2 participants (as suggested by the RAND/UCLA methodology). The three core statements regarding grade, stage and primary site were also discussed (Table 1). The wording of these statements was sometimes adjusted from the original appropriateness statement in response to discussion. For example three statements related to pathology (Quality of pathology reports; Proportion of histopathology reports presented in a synoptic report; Complete synoptic reporting to College of American Pathologist standards) were combined into a single indicator (Structured pathology report).

**Figure 3:**
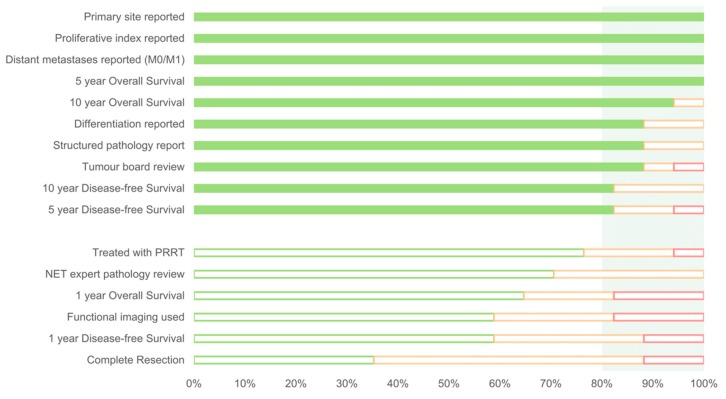
The expert group rated the final Round 2 indicators as appropriate (green), uncertain (orange) or inappropriate (red). The light green shaded area highlights those indicators rated appropriate by more than 80% of the group, thus achieving consensus.

The final statements ranked online in Round 2 are shown in Figure 3 (*n* = 16). The group agreed that stage, grade and primary site were required to robustly interpret all other indicators, in addition to being quality indicators individually. The presence or absence of metastases was chosen to represent stage, whereas both proliferative index and tumour differentiation were required to represent grade.

After the face-to-face discussion in Round 2, the 16 draft indicators were rated online. Those indicators rated “appropriate” by at least 80% of the working group were accepted as the final consensus-derived indicators (*n* = 10; Figure 3 and Table 2). Of note, all of the ‘last chance’ indicators discussed at Round 2 were excluded during this process. The panel noted that proliferative index and differentiation is not required for pheochromocytoma, paraganglioma and medullary thyroid carcinoma.

## 4. Discussion

In the absence of an evidence base, we used a carefully structured inductive multi-stage process to generate a large set of candidates, and then rate and select QPIs by consensus of experts. The process was deliberately multinational, multidisciplinary, and the patient voice was included at every step. The result is 10 consensus NET QPIs that can be trialled to assess NET care.

The consensus NET QPIs might appear generic at first glance, but review of discussion transcripts and notes suggests that the QPIs capture aspects of diagnosis and care that are inherent to NET outcome. Three of the 10 QPIs are pathology-focused, acknowledging the heterogeneity and variable biology of NETs. ‘Proliferative index reported’ and ‘Differentiation reported’ form the basis of grade and determine outcome in most NETs, and ‘Structured pathology report’ acknowledges the need for consistent reporting of these fundamental attributes. NET rarity, heterogeneity and the multidisciplinary nature of NET care is acknowledged in ‘Tumour board review’. The inclusion of ‘Primary site reported’ recognises that different clinical behaviour is observed from different NET primary sites, but also the variable quality of staging of NETs in the metastatic setting, and variable access to NET-specific imaging such as Ga68-DOTA-tate PET CT. In the same way, ‘Distant metastases reported’ acknowledges both the requirement for and quality of radiological staging in most NETs. Finally, the time points of the four survival QPIs were skewed to match the natural history of NETs, recognising the predominantly favourable disease course with 5- and 10-year measures rather than the shorter time frames used in other cancer types.

The rigorous assessment of measurability excluded many potentially important QPIs, leading to limitations in the consensus NET QPIs. There are no QPIs that address the time from first symptom to diagnosis, treatment provision, or follow-up after resection. In these situations, reliable and measurable QPIs could not be agreed. There are no indicators with highly granular outcomes specific to NETs, such as echocardiographic assessment of carcinoid heart disease and there are no outcome measures specific to high grade NETs (e.g., 1-year overall survival). In addition, no indicators relating to the functional aspects of NETs were selected as a QPI. The relevant statements were not considered to be of sufficient applicability to NETs as a whole, although it was recognised that they could be valuable in specific settings and NET subtypes. The functional imaging QPI was rated as appropriate by 60% of the final Round 2 group and, therefore, did not meet the criteria for inclusion as a consensus derived QPI. This potentially reflects both the heterogeneity of NETs (functional imaging is not required for care of some NETs) and access to functional imaging in different health systems. Not all patients with NETs require functional imaging, and the utility of radiotracers varies with grade of NET and over time within individuals with NETs. As a QPI, functional imaging would measure how often functional imaging is used, and whether it is available and utilised in the appropriate setting (e.g., detection of occult primary, pre-operative staging, and for selection of patients for peptide receptor radionuclide therapy). As access to functional imaging becomes more universal, the value of a functional imaging QPI is likely to increase.

The outcome QPIs describe long time frames that are appropriate for the biological behaviour of NETs, but will make actionability difficult due to the delay in seeing the impact of any intervention. This may restrict 10-year survival to use in retrospective comparison, rather than prospective monitoring of ongoing care. Multiple measures of survival were identified as candidates in Round 0 (including overall survival, disease-free survival, progression free survival and disease control rate) but only overall survival and disease-free survival were included in the final 10 indicators. Overall survival is the most easily measured outcome measure, using mortality data, and is important for all NETs. Progression free survival, disease-free survival and disease control rate are less easily measured using routinely collected data in most jurisdictions. The inclusion of disease-free survival as a QPI reflects its importance in measuring outcome in NET patients receiving curative treatment.

The modified RAND/UCLA Delphi method used for NET QPI development is essentially qualitative, and care is needed to avoid introducing bias. For example, the project had been planned to review existing evidence-based indicators from the literature, but we generated draft indicators de novo which is inferior to selection from factors known to be associated with survival (or meaningful patient reported outcomes). Bias could be introduced at each phase of NET QPI development. The statements generated in Round 0 using NGT might reflect the beliefs of participants; careful use of multidisciplinary members, assigned group membership and a method that encouraged participation was used to ameliorate this. The Round 1 questionnaire was moderately arduous (62 statements, with sub-statements in check boxes), which might have introduced bias by retrieving opinions from only the most engaged participants. Participants in Round 2 were able to ‘bring forward’ lower ranked statements for further discussion (as required by the RAND/UCLA method) to address missing parts of the patient journey, or highly aspirational indicators that might not have fared well in Round 1 online assessment. This could also introduce bias, but interestingly, none of these added statements were valued highly enough in Round 2 to make it to the final consensus list. This implies validity for the Round 1 ranking process; only indicators that had been ranked as important AND measurable in Round 1 were finally accepted as the consensus QPIs after Round 2.

It is also interesting to consider the level of expertise indirectly recommended by each consensus NET QPI, and the point on a continuum from generalist through to ‘NET-specific expert’ needed to achieve each QPI. To explain, the Round 2 expert group assessed several statements that required a very high level of NET expert care: such as review in a “NET-specific” tumour board, review by a “NET expert” pathologist, the use of functional imaging; and availability of radionuclide therapy. These four statements did not reach the consensus threshold and were rejected; for example, review by any type of tumour board was deemed acceptable, thus advocating tertiary- but not quaternary-level review. This was thought to facilitate a reasonable standard of care across the three CommNETs countries at this point in time, and might help translation of the QPIs to other countries outside CommNETs. Arguably, these more aspirational indicators should be monitored and assessed for inclusion in future NET QPI sets if the provision of clinical care catches up with the aspirations of the providers.

These QPIs were developed with the aim of measuring care quality across health care systems, but can also provide a guide to individual physicians in their care of people with NETs, particularly the non-outcome based QPIs. For example, at diagnosis, has the primary site been identified and is staging complete? Are grade and pathological differentiation reported in a structured pathology report? If not, imaging and formal pathological review should be requested, with additional imaging and pathological assessment undertaken as required to obtain primary site, stage and grade. Discussion at a multidisciplinary team meeting or tumour board is recommended, and may facilitate obtaining this key information. In this way, the consensus NET QPIs will help improve care of people with NETs and increase the comfort of clinicians caring for people with NETs at an individual level. The next stage of this project will be to measure performance on these QPIs and feed back to providers; this feedback is expected to change clinician behavior by providing both a tacit message of what is required for good care, and by showing each organisation where they are under-performing.

A number of organisations have published management guidelines for NETs that cover aspects of care including diagnosis, imaging, surgical and systemic therapies, and follow up for NETs originating in different anatomical locations [28,29,30,31,32,33,34]. As noted above, the heterogeneity seen in NETs is one of the challenging features in designing QPIs, and similarly there are multiple guidelines for each primary site. The proposed QPIs can be conceptualized as a highly measurable and concise subset of these many guidelines. The molecular make up of NETs and how this impacts on treatment response is an area of active research, and as the understanding of molecular subtypes evolves these will become potential candidate indicators in the future.

This project will now move to trialing of the consensus NET QPIs, initially by application to retrospective registry data. The aim is to understand associations between the NET QPIs that describe diagnosis and investigation, with those that describe outcome. The role of the NET QPIs for international comparison will be assessed. The selection of QPIs is a dynamic process and should be kept under regular review and adapted to changes according to emerging evidence and clinical practice. Considered debate will be undertaken to decide how QPIs are reported to stakeholders.

## 5. Conclusions

This CommNETs project has developed and refined a small set of consensus NET QPIs. These NET QPIs will now be trialed as a method to monitor and improve care for people with NETs and to facilitate international comparison.

## Figures and Tables

**Table 1:** QPI statements assessed in Round 2 and the rationale for further assessment.

**Core statements**
Grade
Stage
Primary site
**Important and measurable statements**
Quality of pathology reports
Pathology involvement in MDM review *
MDM review *
Proportion patients with structural imaging
Proportion of patients with functional imaging in staging
Proportion of histopathology reports presented in a synoptic report
Survival after diagnosis
Complete synoptic reporting to College of American Pathologists standards
**‘Last chance’ statements**
Proportion of patients receiving systemic treatment
Proportion of patients with surgical consultation for consideration of resection
Proportion of patients who receive surgery with curative intent
Proportion of patients getting resection is an important and measurable indicator of NET care quality
Patient reported quality of life
All cases reported to national registry
Proportion of patients with functional symptom control
Proportion of carcinoid patients who have cardiac imaging
Proportion of NET patients diagnosed with carcinoid heart disease (using echocardiogram)

* Multidisciplinary meeting (MDM) was considered the same as tumour board review.

**Table 2:** Final consensus-derived NET QPIs.

Primary site reported
Proliferative index reported *
Distant metastases reported (M0/M1)
5-year overall survival
10-year overall survival
Differentiation reported *
Structured pathology report
Tumour board review
5-year disease-free survival
10-year disease-free survival

* this does not currently apply to pheochromocytoma, paraganglioma and medullary thyroid carcinoma.

## Supplementary Materials

The following are available online at https://www.mdpi.com/2077-0383/8/9/1455/s1: Material S1: Literature Search, Material S2: Participants, Material S3: ‘Round 0’ methodology, Material S4: ‘Round 1’ Online Survey, Material S5: Weighted score, Material S6: Round 2—modified RAND/UCLA Delphi Consensus expert group ranking, Material S7: Round 1 results index.
