# Supplementary material for: Consensus-Derived Quality Performance Indicators for Neuroendocrine Tumour Care
Source: J Clin Med. 2019 Sep 12;8(9):1455. doi: 10.3390/jcm8091455 (PMC6780732; doi:10.3390/jcm8091455)
Supplement: Supplementary file 1 [file jcm-08-01455-s001.pdf]

Consensus-derived quality performance indicators for neuroendocrine tumour care

Supplementary Material

Supplementary Material 1: Literature Search

Pubmed, Medline (Ovid), Embase and Scopus were searched for key terms as follows.

Pubmed: ("performance indicator" OR "quality indicator" OR "quality of care" OR "quality assurance" OR "quality-related process") AND (neuroendocrine OR carcinoid) which reported 22 records, all of which were excluded.

Medline (ovid): ((Carcinoma, Neuroendocrine/ or Neuroendocrine Tumors/) OR (carcinoid.mp. or Carcinoid Tumor/)) AND (Quality Assurance, Health Care/ or Quality Indicators, Health Care/ or quality indicator.mp. or "Quality of Health Care"/) which reported 10 records, all of which were excluded.

Embase: (health care quality/ or quality indicator.mp. OR quality control/) AND (neuroendocrine tumor/ or gastroenteropancreatic neuroendocrine tumor/ OR neuroendocrine tumor.mp. OR carcinoid/ or stomach carcinoid/ or bronchus carcinoid/ or gastrointestinal carcinoid/) which reported 206 records, all of which were excluded.

Scopus: (TITLE-ABS-KEY ("neuroendocrine tumor") OR TITLE-ABS-KEY (carcinoid) OR TITLE-ABS-KEY (neuroendocrine AND tumour) OR TITLE-ABS-KEY (neuroendocrine AND carcinoma) AND TITLE-ABS-KEY (quality AND indicator) OR TITLE-ABS-KEY (quality AND improvement) OR TITLE-ABS-KEY (quality AND care) OR TITLE-ABS- KEY (quality AND indicator) which reported 470 records, all of which were excluded.

Supplementary Material 2: Participants

***Supplementary Table 2.1**. 'Round 0' participants.*

Specialty	Country Australia	New Zealand	Canada	*Total*
Endocrinology	2	2	0	*4*
Medical Oncology	7	1	10	*18*
Nuclear Medicine	3	0	2	*5*
Nurse	0	1	0	*1*
Pathology	0	0	2	*2*
Patient/Patient Advocate	2	0	1	*3*
Pharmacist	0	0	1	*1*
Radiation Oncology	0	0	1	*1*
Radiology	0	1	0	*1*
Researcher	1	1	3	*5*
Surgery	0	2	3	*5*

***Supplementary Table 2.2.** 'Round 1' participants.*

The Round 1 survey was sent to 237 people (CommNETs members n=147; NZ NET multidisciplinary meeting list n=78; and NZ Ministry of Health Cancer Services n=12).

Participants who completed the survey are presented in the table below. Two participants selected multiple specialties in Round 1: Patient/patient advocate/Doctor and Doctor/Researcher. A single specialty is presented for each participant based on the following prioritisation hierarchy: patient/patient advocate, doctor, nurse, researcher, health policy.

Specialty	Country Australia	New Zealand	Canada	*Total*
Doctor	26	19	15	*60*
Nurse	1	1		*2*
Researcher		2		*2*
Patient/patient advocate	2	2	1	*5*
Health Policy		2		*2*

***Supplementary Table 2.3.** 'Round 2' expert working group.*

Specialty	Country Australia	New Zealand	Canada	*Total*
Pathologist		1		1
Surgeon		1		1
Nuclear Medicine	2			2
Medical Oncologist	5	2	3	10
Nurse	1			1
Researcher		1		1
Patient advocate		1		1

Supplementary Material 3: ‘Round 0’ methodology

Candidate indicator generation

For each question, participants generated and wrote down candidates in silence. Then each participant stated their idea to their group, and this was transcribed verbatim by the group facilitator onto a large flipchart, without question or discussion. One idea was shared from each person, continuously circling the group until there were no further ideas. Any new ideas generated by this process were also written verbatim. Then the facilitator led group discussion of each candidate. The wording of each candidate was altered only if recommended by the person who generated the idea. Then the flip chart was placed on a wall, and each group member attached a sticker to select the top five candidates from the work of their group. The five indicators from each group with the most stickers were selected to take forward into the Delphi consensus process.

Candidate indicator curation

Candidates related to the same aspect of the patient journey were organised to create appropriateness statements using a hierarchical structure; a parent statement referred to the major concept (e.g., Survival after diagnosis … …), sometimes followed by sub-statements that further defined the parent statement in a checkbox format (e.g., … … Overall survival, Disease-free survival, Disease control rate, Progression free survival”; and then for this example a further set of checkboxes … … at 1 year, 2 years, 3 years, 4 years, 5 years, 6 years, 7 years, 8 years, 9 years, 10 years from diagnosis). The sub-statements were always descriptors of the parent appropriateness statement, so if the parent statement was not rated as important or measurable, then neither were the accompanying sub-statements. The sub-statements aimed to encourage direct comparison and to reduce the time taken to complete the Round 1 survey. In some cases, a parent statement was created de novo to improve the structure of the questionnaire (e.g., Measurement of quality of life…) to allow grouping of similar sub-statements that could be compared using checkboxes (e.g., Which of the following measures of quality of life … … Quality of life measured at diagnosis, Performance status measured at diagnosis, Quality of life monitoring, Regular assessment of symptom control). After this curation, appropriateness statements and sub-statements were taken forward to Round 1 for rating (see Figure 1 and Supplementary Material 4).

Supplementary Material 4: ‘Round 1’ Online Survey

Online Survey

Survey Monkey® was used to present appropriateness statements and record ratings and feedback. The first page collected information about the participant. An introductory page included links to short descriptions of the background, method, and instructions. A glossary was provided by hyperlink from the first time a defined word was used in the survey. The order of appropriateness statements was similar to the chronology of the patient journey. Similar indicators were grouped on the same page. Participants separately rated the *importance* of each statement, and the *measurability* of each statement.

The survey was sent to all CommNETs members; NZ NET multidisciplinary meeting list; and NZ Ministry of Health Cancer Services (Supplementary Material 2). Some participants forwarded the survey link to their own NET clinical communities. From the time the initial survey invitation was circulated, each person received at least two email reminders before closure of the survey.

Below are the statements as presented in the ‘Round 1’ survey.

**Statement number**	**Statement**	**Answer options for sub-statements (checkbox format)**
1	Country (required)	
2	State/Province (required)	
3	Primary Professional Role (required)	Doctor
		Nurse
		Another clinical role
		Researcher
		Patient/Patient advocate
		Health Policy
		Other (please specify)
4	Name (preferred, but optional)	
5	Email Address (preferred, but optional)	
6	NET primary site is required to robustly interpret each indicator of NET care quality	
7	NET tumour stage is required to robustly interpret each indicator of NET care quality	
8	NET tumour grade is required to robustly interpret each indicator of NET care quality	
9	Do you have any additional comments? (not required)	
10	Survival after diagnosis is an important and measurable indicator of NET care quality	
11	Which measures of survival are the most important indicators of NET care quality? Please choose two.	Overall survival
		Disease-free survival
		Disease control rate
		Progression free survival
12	Which measures of survival are the most measurable indicators of NET care quality? Please choose two.	Overall survival
		Disease-free survival
		Disease control rate
		Progression free survival
13	Which time points are most appropriate to measure survival? Please choose three.	1 year from diagnosis
		2 years from diagnosis
		3 years from diagnosis
		4 years from diagnosis
		5 years from diagnosis
		6 years from diagnosis
		7 years from diagnosis
		8 years from diagnosis
		9 years from diagnosis
		10 years from diagnosis
		15 years from diagnosis
		20 years from diagnosis
14	Documentation of cause of death for patients who die from NETs is an important and measurable indicator of NET care quality	
15	Do you have any additional comments? (not required)	
16	Patient reported quality of life is an important and measurable indicator of NET care quality	

17	Which aspects of patient reported quality of life are the most important indicators of NET care quality? Please choose three.	Physical wellbeing
		Financial toxicity
		Emotional wellbeing
		Functional status
		Symptoms
		Satisfaction of care
		Disability
		Edmonton Symptom Assessment Scale monitoring
		Patient reported outcome monitoring
		Toxicity of treatment measured by patient reported outcomes
18	Which aspects of patient reported quality of life are the most measurable indicators of NET care quality? Please choose three.	Physical wellbeing
		Financial toxicity
		Emotional wellbeing
		Functional status
		Symptoms
		Satisfaction of care
		Disability
		Edmonton Symptom Assessment Scale monitoring
		Patient reported outcome monitoring
		Toxicity of treatment measured by patient reported outcomes
19	Do you have any additional comments? (not required)	
20	Surrogates for quality of life are important and measurable indicators of NET care quality	
21	Which of the following surrogates for quality of life is the most important indicator of NET care quality? Please choose one.	ED attendance
		Hospital admission
		Unplanned hospital admissions
22	Which of the following surrogates for quality of life is the most measurable indicator of NET care quality? Please choose one.	ED attendance
		Hospital admission
		Unplanned hospital admissions
23	Which time points are the most appropriate to measure quality of life (including patient reported quality of life and surrogates for quality of life)? Please choose three.	At diagnosis
		1 year from diagnosis
		2 years from diagnosis
		3 years from diagnosis
		4 years from diagnosis
		5 years from diagnosis
		6 years from diagnosis
		7 years from diagnosis
		8 years from diagnosis
		9 years from diagnosis
		10 years from diagnosis
		15 years from diagnosis
		20 years from diagnosis

24	Do you have any additional comments? (not required)	
25	Measurement of quality of life is an important and measurable indicator of NET care quality	
26	Which of the following measures of quality of life are the most important indicators of NET care quality? Please choose two.	Quality of life measured at diagnosis
		Performance status measured at diagnosis (e.g. ECOG performance score or Karnofsky score)
		Quality of life monitoring
		Regular assessment of symptom control
27	Which of the following measures of quality of life are the most measurable indicators of NET care quality? Please choose two.	Quality of life measured at diagnosis
		Performance status measured at diagnosis (e.g. ECOG performance score or Karnofsky score)
		Quality of life monitoring
		Regular assessment of symptom control
28	Proportion of patients who report they are NET symptom-free at 18 months using symptom questionnaire is an important and measurable indicator of NET care quality	
29	Proportion of patients with functional symptom control is an important and measurable indicator of NET care quality	
30	Proportion of patients with non-functional symptom control is an important and measurable indicator of NET care quality	
31	Do you have any additional comments? (not required)	
32	Proportion of NET patients diagnosed with carcinoid heart disease (using echocardiogram) is an important and measurable indicator of NET care quality	
33	Proportion of carcinoid patients who have cardiac imaging is an important and measurable indicator of NET care quality	
34	Which time points are the most appropriate to look for carcinoid heart disease? Please choose three.	At diagnosis
		1 year from diagnosis
		2 years from diagnosis
		3 years from diagnosis
		4 years from diagnosis
		5 years from diagnosis
		6 years from diagnosis
		7 years from diagnosis
		8 years from diagnosis
		9 years from diagnosis
		10 years from diagnosis
		15 years from diagnosis
		20 years from diagnosis
35	Do you have any additional comments? (not required)	
36	Proportion of patients receiving a needs assessment is an important and measurable indicator of NET care quality	
37	Proportion of patients receiving a care plan is an important and measurable indicator of NET care quality	
38	Proportion of patients receiving a treatment summary is an important and measurable indicator of NET care quality	
39	Proportion of patients offered allied health services is an important and measurable indicator of NET care quality	

40	Proportion of patients offered patient support groups is an important and measurable indicator of NET care quality	
41	Do you have any additional comments? (not required)	
42	The time interval until treatment is started is an important and measurable indicator of NET care quality	
43	Which of the following time intervals, are the most important indicators of NET care quality? Please choose two.	Time from diagnosis to first treatment
		Time from diagnosis to treatment plan
		Time from diagnosis to decision to treat
		Time from decision to treat to definitive treatment
44	Which of the following time intervals, are the most measurable indicators of NET care quality? Please choose two.	Time from diagnosis to first treatment
		Time from diagnosis to treatment plan
		Time from diagnosis to decision to treat
		Time from decision to treat to definitive treatment
45	The time interval from first doctors visit with symptoms of NET, to diagnosis of NET is an important and measurable indicator of NET care quality	
46	Which of the following time intervals are the most important indicators of NET care quality? Please choose three.	Time from presentation to NET diagnosis
		Time from presentation to any specialist referral
		Time from presentation to NET specialist referral
		Time from symptoms to NET diagnosis
		Time from initial symptoms to any specialist referral
		Time with "symptoms misdiagnosis"
		Number of GP visits
		Number of health assessments
		Number of medical contacts
		Other (please specify)
47	Which of the following time intervals are the most measurable indicators of NET care quality? Please choose three.	Time from presentation to NET diagnosis
		Time from presentation to any specialist referral
		Time from presentation to NET specialist referral
		Time from symptoms to NET diagnosis
		Time from initial symptoms to any specialist referral
		Time with "symptoms misdiagnosis"
		Number of GP visits
		Number of health assessments
		Number of medical contacts
48	Do you have any additional comments? (not required)	
49	Proportion of patients with surgical consultation for consideration of resection is an important and measurable indicator of NET care quality	
50	Proportion of patients getting resection is an important and measurable indicator of NET care quality	
51	Proportion of patients who receive surgery with curative intent is an important and measurable indicator of NET care quality	
52	Proportion patients who receive non-curative surgery is an important and measurable indicator of NET care quality	
53	Do you have any additional comments? (not required)	

54	Assessment of surgical quality is an important and measurable indicator of NET care quality	
55	Which of the following measures of surgical quality are the most important indicators of NET care quality? Please choose two.	Median length of stay following primary resection
		Median length of stay following metastatic resection
		Mortality at 30 days after surgery
		Mortality at 90 days after surgery
56	Which of the following measures of surgical quality are the most measurable indicators of NET care quality? Please choose two.	Median length of stay following primary resection
		Median length of stay following metastatic resection
		Mortality at 30 days after surgery
		Mortality at 90 days after surgery
57	The quality of pre-operative assessment is an important and measurable indicator of NET care quality	
58	Which pre-operative assessment is the most important indicator of NET care quality? Please choose one.	Appropriate clinical assessment
		Appropriate functional assessment
		Appropriate radiological assessment
59	Which pre-operative assessment is the most measurable indicator of NET care quality? Please choose one.	Appropriate clinical assessment
		Appropriate functional assessment
		Appropriate radiological assessment
60	Complete pre-operative TNM staging is an important and measurable indicator of NET care quality	
61	Complete post-operative TNM staging is an important and measurable indicator of NET care quality	
62	Do you have any additional comments? (not required)	
63	Proportion of patients who see who see a NET specialist is an important and measurable indicator of NET care quality	
64	Review by specialist (by any means including virtual review) at least annually is an important and measurable indicator of NET care quality	
65	MDM review is an important and measurable indicator of NET care quality	
66	Which of the following measures of multidisciplinary care are the most important indicators of NET care quality? Please choose three.	Proportion of cases discussed at MDM
		Proportion patients who are discussed at MDM at diagnosis
		Evaluation in a multi-disciplinary clinic prior to first treatment
		Evaluation in a MDM prior to first treatment
		Case discussed at MDM that include a diagnostic radiologist
		MDM issued grade and stage statement (TNM)
		Clear documentation of MDM discussion with all stakeholders
		Communication of tumour board consensus
		Proportion patients who are discussed at MDM with any new recurrence
67	Which of the following measures of multidisciplinary care are the most measurable indicators of NET care quality? Please choose three.	Proportion of cases discussed at MDM
		Proportion patients who are discussed at MDM at diagnosis
		Evaluation in a multi-disciplinary clinic prior to first treatment
		Evaluation in a MDM prior to first treatment
		Case discussed at MDM that include a diagnostic radiologist
		MDM issued grade and stage statement (TNM)
		Clear documentation of MDM discussion with all stakeholders
		Communication of tumour board consensus

		Proportion patients who are discussed at MDM with any new recurrence
68	Pathology involvement in MDM review is an important and measurable indicator of NET care quality	
69	Which of the following measures of pathology involvement in MDM review is the most important indicator of NET care quality? Please choose one.	Proportion discussed at a MDM that includes a pathologist
		Proportion of cases with histology reviewed by a NET expert pathologist”
		Review by a specialist gastrointestinal “GI” pathologist
70	Which of the following measures of pathology involvement in MDM review is the most measurable indicator of NET care quality? Please choose one.	Proportion discussed at a MDM that includes a pathologist
		Proportion of cases with histology reviewed by a NET expert pathologist
		Review by a specialist gastrointestinal “GI” pathologist
71	Do you have any additional comments? (not required)	
72	The quality of pathology reports is an important and measurable indicator of NET care quality	
73	Which of the following pathology reporting measures are the most important indicators of NET care quality? Please choose three.	pTNM stage
		Margin
		Node
		Tumour size
		Depth of invasion
		Ki-67
		Mitotic rate
		Grade
		IHC (synaptophysin/chromogranin)
		Necrosis
		Differentiation
		LVI
		Site
74	Which of the following pathology reporting measures are the most measurable indicators of NET care quality? Please choose three.	pTNM stage
		Margin
		Node
		Tumour size
		Depth of invasion
		Ki-67
		Mitotic rate
		Grade
		IHC (synaptophysin/chromogranin)
		Necrosis
		Differentiation
		LVI
		Site
75	Proportion of histopathology reports presented in a synoptic report is an important and measurable indicator of NET care quality	

76	Complete synoptic reporting to College of American Pathologists standards is an important and measurable indicator of NET care quality	
77	A comprehensive but non-synoptic report is an important and measurable indicator of NET care quality	
78	Do you have any additional comments? (not required)	
79	Pathological classification based on ENETS staging is an important and measurable indicator of NET care quality	
80	Proportion of NET patients with TNM staging at diagnosis is an important and measurable indicator of NET care quality	
81	The use of which of the following TNM staging guidelines is the most important indicator of NET care quality? Please choose one.	ENET guidelines
		UICC guidelines
82	The use of which of the following TNM staging guidelines is the most measurable indicator of NET care quality? Please choose one.	ENET guidelines
		UICC guidelines
83	Do you have any additional comments? (not required)	
84	Proportion of patients with functional imaging in staging is an important and measurable indicator of NET care quality	
85	If staging functional imaging is used, the use of which scans are the most important indicators of NET care quality? Please choose two.	Any peptide receptor imaging
		FDG scan
		Ga68 DOTA tate PET
		Octreoscan
86	If staging functional imaging is used, the use of which scans are the most measurable indicators of NET care quality? Please choose two.	Any peptide receptor imaging
		FDG scan
		Ga68 DOTA tate PET
		Octreoscan
87	Proportion patients with structural imaging is an important and measurable indicator of NET care quality	
88	If structural imaging is used, which of the following are the most important indicators of NET care quality? Please choose three.	Contrast CT scan
		Liver MRI
		Echocardiogram
		Triphasic CT prior to surgical resection
		Triphasic MRI prior to surgical resection
		Endoscopic Ultrasound
89	If structural imaging is used, which of the following are the most measurable indicators of NET care quality? Please choose three.	Contrast CT scan
		Liver MRI
		Echocardiogram
		Triphasic CT prior to surgical resection
		Triphasic MRI prior to surgical resection
		Endoscopic Ultrasound
90	Proportion of radiology synoptic reporting is an important and measurable indicator of NET care quality	
91	Time from diagnosis to completion of staging is an important and measurable indicator of NET care quality	
92	Appropriate radiological staging is an important and measurable indicator of NET care quality	
93	Do you have any additional comments? (not required)	

94	Proportion who get biochemical functional workup is an important and measurable indicator of NET care quality	
95	If a biochemical workup is completed, which of the following are the most important indicators of NET care quality? Please choose three.	CgA level
		CgA with appropriate dietary restriction
		5HIAA level
		Urinary 5HIAA level with appropriate dietary restriction
		Calcium level
		pNETs baseline biochemistry
		Appropriate biochemical staging
		Ongoing biochemical monitoring
		Proportion with functional status assessed (biochemistry)
96	If a biochemical workup is completed, which of the following are the most measurable indicators of NET care quality? Please choose three.	CgA level
		CgA with appropriate dietary restriction
		5HIAA level
		Urinary 5HIAA level with appropriate dietary restriction
		Calcium level
		pNETs baseline biochemistry
		Appropriate biochemical staging
		Ongoing biochemical monitoring
		Proportion with functional status assessed (biochemistry)
97	Do you have any additional comments? (not required)	
98	Recording of history is an important and measurable indicator of NET care quality	
99	If history is recorded, which of the following are the most important indicators of NET care quality? Please select three.	Comprehensive history
		Family history
		Targeted family history
		Functional symptoms
		Comorbidities
		Weight loss
100	If history is recorded, which of the following are the most measurable indicators of NET care quality? Please select three.	Comprehensive history
		Family history
		Targeted family history
		Functional symptoms
		Comorbidities
		Weight loss
101	Percent patients with clearly elicited and documented secretory symptoms is an important and measurable indicator of NET care quality	
102	Comprehensive assessment of baseline symptoms and symptom history is an important and measurable indicator of NET care quality	
103	Consideration of concurrent endocrine issues is an important and measurable indicator of NET care quality	
104	Do you have any additional comments? (not required)	
105	Recording of clinical examination details is an important and measurable indicator of NET care quality	

106	Which of the following examinations are the most important indicators of NET care quality? Please choose two.	ECOG
		Weight
		Cardiac examination
		Comprehensive physical exam
107	Which of the following examinations are the most measurable indicators of NET care quality? Please choose two.	ECOG
		Weight
		Cardiac examination
		Comprehensive physical exam
108	Do you have any additional comments? (not required)	
109	Type of presentation (e.g. incidental, symptoms, etc) is an important and measurable indicator of NET care quality	
110	Which of the following presentation types is the most important indicator of NET care quality? Please choose one.	Acute emergent presentation
		CT as incidental finding
		Familial screening or other screening programs (c-scope, g-scope)
111	Which of the following presentation types is the most measurable indicator of NET care quality? Please choose one.	Acute emergent presentation
		CT as incidental finding
		Familial screening or other screening programs (c-scope, g-scope)
112	Do you have any additional comments? (not required)	
113	Proportion of patients offered a clinical trial is an important and measurable indicator of NET care quality	
114	Proportion of patients entered into a clinical trial is an important and measurable indicator of NET care quality	
115	Proportion of patients whose tissue is banked is an important and measurable indicator of NET care quality	
116	Do you have any additional comments? (not required)	
117	All cases reported to national registry is an important and measurable indicator of NET care quality	
118	Registry enrolment at diagnosis is an important and measurable indicator of NET care quality	
119	Do you have any additional comments? (not required)	
120	The proportion of patients receiving systemic treatment is an important and measurable indicator of NET care quality	
121	Which of the following statements about systemic therapy are the most important indicators of NET care quality? Please choose three.	Proportion of metastatic NET patients who are receiving targeted systemic therapy
		Proportion of metastatic NET patients who are receiving chemotherapy
		Proportion of metastatic NET patients who are receiving PRRT
		Proportion of metastatic NET patients who are receiving SSA
		Proportion of patients who have had three or more lines of therapy
		Proportion of metastatic patients who receive 1st, 2nd, 3rd systemic therapy
		Proportion of patients with liver directed therapy
		Proportion of patients with locoregional therapy
122	Which of the following statements about systemic therapy are the most measurable indicators of NET care quality? Please choose three.	Proportion of metastatic NET patients who are receiving targeted systemic therapy
		Proportion of metastatic NET patients who are receiving chemotherapy
		Proportion of metastatic NET patients who are receiving PRRT

		Proportion of metastatic NET patients who are receiving SSA
		Proportion of patients who have had three or more lines of therapy
		Proportion of metastatic patients who receive 1st, 2nd, 3rd systemic therapy
		Proportion of patients with liver directed therapy
		Proportion of patients with locoregional therapy
123	Which of the following statements about choice of systemic therapy is the most important indicator of NET care quality? Please choose one.	Proportion of patients with treatment by guideline
		Proportion of patients who had access to proven therapy
		Proportion of patients offered all globally proven therapies and analysed by each therapy
124	Which of the following statements about choice of systemic therapy is the most measurable indicator of NET care quality? Please choose one.	Proportion of patients with treatment by guideline
		Proportion of patients who had access to proven therapy
		Proportion of patients offered all globally proven therapies and analysed by each therapy
125	Do you have any additional comments? (not required)	
126	Ongoing radiological monitoring is an important and measurable indicator of NET care quality	
127	Imaging events after curative surgery is an important and measurable indicator of NET care quality	
128	Proportion of patients with bowel obstruction is an important and measurable indicator of NET care quality	
129	Which time points are the most appropriate to measure this? Please choose three.	At diagnosis
		1 year from diagnosis
		2 years from diagnosis
		3 years from diagnosis
		4 years from diagnosis
		5 years from diagnosis
		6 years from diagnosis
		7 years from diagnosis
		8 years from diagnosis
		9 years from diagnosis
		10 years from diagnosis
		15 years from diagnosis
		20 years from diagnosis
130	Do you have any additional comments? (not required)	
131	The proportion of patients who have unknown primary site of disease is an important and measurable indicator of NET care quality	
132	Proportion of patients diagnosed by each specialty is an important and measurable indicator of NET care quality	
133	Proportion of patients with metastases at initial diagnosis is an important and measurable indicator of NET care quality	
134	Do you have any additional comments? (not required)	
135	Thank you very much for the time taken to complete this survey, your input is greatly appreciated! Do you have any final comments? (not required)	

Supplementary Material 5: Weighted score

Participants were asked to rate appropriateness statements on a Likert scale of 1 = Highly inappropriate to 9 = Highly appropriate (5 = Uncertain).

Rating score	Weighted multiplier
1	-4
2	-3
3	-2
4	-1
5	0
6	1
7	2
8	3
9	4

Each score was weighted based on the number of responses; weighted multiplier * number of responses = weighted value.

The positive weighted values (corresponding to rating score of 6-9) were summed and divided by the number of responses.

To adjust for the total number of responses, this was multiplied by the ratio of positive (rating score 6-9): total number of responses, excluding uncertain ratings (“5”).

Using a weighted average gave a sense of the level of agreement within the group, i.e. whether there were a high number of both high and low ratings, or whether there was strong consensus for an average rating.

Supplementary Material 6: Round 2 – modified RAND/UCLA Delphi Consensus expert group ranking

As required by the modified Delphi method, a small expert group (see Supplementary Material 2.3) met to discuss appropriateness statements that had been top ranked in the Round 1 survey, and select a subset of final indicators by consensus. A reading pack had been distributed that included a reminder of the background to the project, methods of analysis and data presentation, and a summary of results for all statements The summary included a single bar chart for each statement showing the weighted appropriateness, with the positive and negative response presented separately for each statement. Round 1 responder comments from free-text survey fields were presented next to each relevant statement.

It was affirmed that disagreement was acceptable, the group was not forced to agree, but only statements with consensus would progress as NET QPIs. The videoconference was recorded by ZOOM, transcribed and combined with notes from three people taken during the meeting (KP, BL, BW). Following the meeting, a rating form was circulated online for ranking the draft indicators (n=16) by the expert group as appropriate, uncertain, or inappropriate. Each indicator was presented alongside the relevant discussion points raised during the teleconference. A comment field was offered after every indicator to ensure the working group were able to communicate their opinion clearly and ensure accurate feedback was received on the proposed indicator list. Final NET QPIs were chosen using a consensus threshold of 80%, as utilised in the previous CommNETs Delphi process.

Supplementary Material 7: Round 1 results index

Asterisk (*) indicates statements which were followed by sub-statements in a checkbox format.

Appropriateness Statement Number	Statement
Q72.	The quality of pathology reports*
Q16.	Patient reported quality of life*
Q68.	Pathology involvement in MDM review*
Q65.	MDM review*
Q87.	Proportion patients with structural imaging*
Q92.	Appropriate radiological staging
Q84.	Proportion of patients with functional imaging in staging*
Q75.	Proportion of histopathology reports presented in a synoptic report
Q10.	Survival after diagnosis*
Q101.	Percent patients with clearly elicited and documented secretory symptoms
Q117.	All cases reported to national registry
Q63.	Proportion of patients who see who see a NET specialist
Q118.	Registry enrolment at diagnosis
Q126.	Ongoing radiological monitoring
Q76.	Complete synoptic reporting to College of American Pathologists standards
Q25.	Measurement of quality of life*
Q29.	Proportion of patients with functional symptom control
Q102.	Comprehensive assessment of baseline symptoms and symptom history
Q33.	Proportion of carcinoid patients who have cardiac imaging*
Q94.	Proportion who get biochemical functional workup*
Q98.	Recording of history*
Q54.	Assessment of surgical quality*
Q79.	Pathological classification based on ENETS staging
Q61.	Complete post-operative TNM staging
Q14.	Documentation of cause of death for patients who die from NETs
Q60.	Complete pre-operative TNM staging
Q90.	Proportion of radiology synoptic reporting
Q103.	Consideration of concurrent endocrine issues

Q127.	Imaging events after curative surgery
Q57.	The quality of pre-operative assessment*
Q32.	Proportion of NET patients diagnosed with carcinoid heart disease (using echocardiogram)
Q80.	Proportion of NET patients with TNM staging at diagnosis*
Q45.	The time interval from first doctors visit with symptoms of NET, to diagnosis of NET*
Q120.	The proportion of patients receiving systemic treatment*
Q113.	Proportion of patients offered a clinical trial
Q40.	Proportion of patients offered patient support groups
Q91.	Time from diagnosis to completion of staging
Q49.	Proportion of patients with surgical consultation for consideration of resection
Q64.	Review by specialist (by any means including virtual review) at least annually
Q38.	Proportion of patients receiving a treatment summary
Q133.	Proportion of patients with metastases at initial diagnosis
Q28.	Proportion of patients who report they are NET symptom-free at 18 months using symptom questionnaire
Q30.	Proportion of patients with non-functional symptom control
Q39.	Proportion of patients offered allied health services
Q37.	Proportion of patients receiving a care plan
Q51.	Proportion of patients who receive surgery with curative intent
Q36.	Proportion of patients receiving a needs assessment
Q114.	Proportion of patients entered into a clinical trial
Q105.	Recording of clinical examination details*
Q42.	The time interval until treatment is started*
Q20.	Surrogates for quality of life are important and measurable indicators of NET care quality*
Q115.	Proportion of patients whose tissue is banked
Q128.	Proportion of patients with bowel obstruction*
Q52.	Proportion patients who receive non-curative surgery
Q131.	The proportion of patients who have unknown primary site of disease
Q109.	Type of presentation (e.g. incidental, symptoms, etc)*
Q50.	Proportion of patients getting resection
Q77.	A comprehensive but non-synoptic report
Q132.	Proportion of patients diagnosed by each specialty
